# Rare Disease: Cardiac Risk Assessment With MRI in Patients With Myotonic Dystrophy Type 1

**DOI:** 10.3389/fneur.2020.00192

**Published:** 2020-03-19

**Authors:** Marco Alì, Caterina Beatrice Monti, Luca Melazzini, Rosanna Cardani, Barbara Fossati, Michele Cavalli, Kelvin Chow, Francesco Secchi, Giovanni Meola, Francesco Sardanelli

**Affiliations:** ^1^Unit of Diagnostic Imaging and Stereotactic Radiosurgery, C.D.I. Centro Diagnostico Italiano S.p.A., Milan, Italy; ^2^Unit of Radiology, IRCCS Policlinico San Donato, San Donato Milanese, Italy; ^3^PhD Course in Integrative Biomedical Research, Department of Biomedical Sciences for Health, University of Milan, Milan, Italy; ^4^Laboratory of Muscle Histopathology and Molecular Biology, IRCCS Policlinico San Donato, San Donato Milanese, Italy; ^5^Unit of Neurology, IRCCS Policlinico San Donato, San Donato Milanese, Italy; ^6^Department of Neurorehabilitation Sciences, Casa di Cura del Policlinico, Milan, Italy; ^7^Postgraduate School in Neurology, University of Milan, Milan, Italy; ^8^Siemens Medical Solutions USA Inc., Chicago, IL, United States; ^9^Department of Biomedical Sciences for Health, University of Milan, San Donato Milanese, Italy

**Keywords:** myotonic dystrophy, cardiac magnetic resonance, myocardial strain imaging, extracellular volume, cardiac fibrosis

## Abstract

**Introduction:** To evaluate myocardial strain and extracellular volume in myotonic dystrophy type 1 (DM1) patients as potential imaging biomarkers of subclinical cardiac pathology.

**Materials and methods:** We retrospectively analyzed 9 DM1 patients without apparent cardiac disease who had undergone cardiac magnetic resonance at our center. Patients were age- and sex-matched with healthy controls. The Mann-Whitney U test was used to compare cardiac strain between the two groups. The *t*-test was used to compare the extracellular volume obtained in DM1 patients with that in healthy subject. Spearman's ρ was used for studying the associations among imaging parameters.

**Results:** Global cardiac strain (median −19.1%; IQR −20.5%, −16.5%) in DM1 patients was lower (*p* = 0.011) than that in controls (median−21.7%; IQR−22.7%,-21.3%). Global extracellular volume in DM1 patients (median 32.3%; IQR 29.3%,36.8%) was significantly (*p* = 0.008) higher than that reported in literature in healthy subjects (median 25.6%; IQR 19.9%,31.9%). Global cardiac strain showed a strong, positive correlation with septal strain (ρ = 0.767, *p* = 0.016) and with both global (ρ = 0.733 *p* = 0.025) and septal extracellular volume (ρ = 0.767, *p* = 0.016).

**Discussion:** The increase in cardiac extracellular volume and decrease in strain are signs of early cardiac pathology in DM1. Physicians dealing with DM1 may take into consideration cardiac magnetic resonance as a screening tool to identify early cardiac involvement in this condition.

## Introduction

Myotonic dystrophy type 1 (DM1) is a dominantly inherited neuromuscular rare disorder caused by a mutation (CTG repeat expansion) in the the *Dystrophia Myotonic Protein Kinase* gene that affects 1:8,000 people worldwide (1–4). Cardiac involvement in DM1 occurs in up to 80% of the cases (5, 6), and represents the second most common cause of death in this population, straight after respiratory failure (7). According to current recommendations (8), DM1 patients should be periodically screened for cardiac disease with electrocardiography and echocardiography.

Autoptic examinations of DM1 hearts revealed fibrous tissue infiltration in the conduction system (9) which may cause arrhythmias. However, electrocardiography detects only manifest arrhythmias related to large patches of localized fibrosis, while echocardiography may be hindered by bad acoustic window quality or motion impairment in severe patients.

A viable alternative to these techniques is cardiac magnetic resonance (CMR), which allows to assess both localized and diffuse fibrosis through the analysis of late gadolinium enhancement (LGE) and the calculation of extracellular volume (ECV) with T1-mapping (10, 11) even before the onset of clinical manifestations of heart arrhythmias. Moreover, CMR allows for the calculation of functional parameters such as cardiac strain, an indicator of cardiac contractility, which can be impaired when the myocardium is fibrotic or degenerated (12). Cardiac strain can also be obtained through echocardiography (13, 14) and serve as a predictor of cardiovascular events in asymptomatic DM1 patients (15). However, a study by Khan et al. (16) showed that strain parameters obtained at CMR are more reliable and reproducible than those obtained at echocardiography due to high dependence on the operator and lower acoustic window quality of the latter technique compared to the former.

Few studies have assessed DM1 cardiac involvement using CMR. Petri et al. (17) and Turkbey et al. (18) reported LGE and T1 alterations in patients with advanced cardiac disease. Hermans et al. (19) observed the presence of subclinical cardiomyopathy in DM1 patients through LGE evaluation, stating that their cardiac involvement may be overlooked if screened with electrocardiography alone. However, Petri et al. (17) showed that LGE is only detectable in case of focal fibrosis, while ECV may identify diffuse fibrosis, which may be present in patients at earlier stages of cardiac involvement, potentially preceding localized fibrosis.

Evidence of subclinical cardiac pathology in asymptomatic DM1 patients come also from basic research studies (20–22). Recently, Valaperta et al. (20) found that blood levels of circulating cardiac Troponin T (cTnT), a serological biomarker of cardiac injury, were significantly higher in asymptomatic DM1 patients compared to healthy subjects. The authors observed that the rise in circulating cTnT was more likely due to subclinical myocardial damage, which had not been detected by conventional measures, rather than being caused by a release of cTnT from injured skeletal muscle into the blood pool, as observed in other neuromuscular diseases (20–22).

In light of these considerations, the aim of our study was to evaluate ECV and strain on CMR in DM1 patient and appraise whether they could be viable imaging-biomarkers of subclinical cardiac pathology. Secondly, we evaluated if there were some associations between imaging parameters and data stemming from clinical assessment and laboratory testing.

## Materials and Methods

### Study Design and Population

The local Ethics Committee approved this study (Ethics Committee of San Raffaele University Hospital; protocol code CardioRetro; approved on March 9th, 2017). This research received no specific grant from any funding agency in the public, commercial or not-for-profit sectors. Due to the retrospective nature of this study specific informed consent was waived.

We retrospectively analyzed images of asymptomatic DM1 patients who had undergone a CMR examination as screening test for early cardiac involvement at our cardiovascular referral center (IRCCS Policlinico San Donato, University Hospital) between November 2014 and July 2016. Three out of nine patients were taking mexiletine at the time of the examination. Additional inclusion criteria were:

- Diagnosis of DM1 through genetic testing, based upon the clinical diagnostic criteria set by the International Consortium for Myotonic Dystrophy (23); DM1 genotyping was performed on genomic DNA extracted from peripheral blood leukocytes (24).
- Presence of a complete set of short-axis cine sequences.
- Presence of both native and post-contrast T1 maps.

We excluded patients who had a low (≤50%) ejection fraction, or who presented overt symptoms or signs of cardiac pathology and thus would not represent the ideal population for screening early cardiac damage.

Each patient was age- and sex-matched with a healthy control to compare the strain analysis to that of patients. Controls were chosen among subjects who had been referred to CMR for a more thorough investigation of isolated supraventricular extrasystoles. Such individuals had a negative CMR examination and did not present significant extrasystoles during the examination, thus yielding excellent image quality. None of our controls had been referred for ablation.

### Neuromuscular Assessment and Serum Biomarkers

According to clinical practice, as a part of their routine management, DM1 patients had undergone a baseline and routine clinical neuromuscular examination. Single muscular group and global strength were manually quantified according to the medical research council (MRC) scale (25), which assesses seven muscular groups, grading strength from 0 to 5 in each one, for a score total of 130.

Global muscular impairment in DM1 patients was also evaluated using the muscular impairment rating scale (MIRS) (26), which grades global motor impairment from 5 to 0, which correspond, respectively to the least and most functional impairment.

Moreover, DM1 patients had undergone routine blood tests which included genetic test.

### Cardiological Assessment

According to clinical practice, all DM1 patients had undergone a 12-lead baseline electrocardiogram (EKG) and a 24-h Holter-EKG to detect potential cardiac involvement. The duration of QRS complex, PR, QTc interval, and eventual bouts of atrial fibrillation were also registered.

A transthoracic 2D-echocardiogram had been performed in DM1 patients, and data regarding the ejection fraction was collected.

### Image Assessment

All CMR examinations were performed using a 1.5-T Magnetom Aera unit (Siemens Medical Solutions, Erlangen, Germany), with 45-mT/m gradient power. Examinations were performed using a 12-channel surface phased-array coil, placed over the thorax of the patient in supine position. Image acquisition was electrocardiographically gated.

Every CMR study included a complete set of short-axis (from base to apex) cine images, using an electrocardiographically triggered steady-state free precession pulse sequence acquired with the following technical parameters: time of repetition (TR)/time of echo (TE) 4.0/1.5 ms; flip angle 80°; slice thickness 8 mm; time resolution 45 ms; mean acquisition time 14 ± 4 s (mean ± standard deviation), number of phases 30, in-plane pixel size 1.40 × 1.80 mm^2^.

A native MOLLI sequence with 5(3)3 protocol was acquired at basal, mid, and apical-ventricular level in short-axis view in systole, by starting the data acquisition at an individually adapted trigger time. The MOLLI sequence included motion correction and subsequent automatic generation of T1 maps. MOLLI sequences were acquired with the following parameters: TE/TR 1.12 ms/2.8 ms; flip angle 35°; bandwidth 1,085 Hz/pixel; in-plane pixel size 1.40 × 1.80 mm^2^, parallel imaging factor 2.

Using softwares from the Medis suite (Medis Medical Imaging Systems, Leiden, The Netherlands), the endocardium and epicardium of the left ventricle were manually segmented on cine images. Circumferential strain (CS) was subsequently calculated both globally and for each cardiac segment; Controls CMR examinations included cine evaluation with the same sequence used for patients.

Left ventricle global ECV was calculated from T1 values of different, manually traced, regions of interest on different cardiac regions (namely anterior, lateral, posterior and septal), at LV mid-level, in native and post-contrast T1 maps, considering patient hematocrit values. Since ECV lacks a reference range for healthy subjects, the Society of Cardiovascular Magnetic Resonance recommends comparing patient ECV values with those from previous studies where the same system was used (27). Therefore, the ECV obtained from DM1 patients was compared with that of healthy subjects reported in the meta-analysis by Sardanelli et al. (28).

### Statistical Analysis

Normal data distributions were reported as mean ± standard deviation, while non-normal data distributions were reported as median and interquartile range (IQR). The minimum and the maximum value of each distribution were also reported.

Differences between independent distributions were evaluated using Mann-Whitney U test, while correlations were evaluated using Spearman's ρ. The *t*-test was used to compare the global ECV obtained in our DM1 patients with that published in literature by Sardanelli et al. (28) in healthy subjects.

Statistical analysis was performed using SPSS (IBM SPSS Inc., Chicago, IL, USA), and *p*-values lower than 0.05 were considered as significant.

## Results

### Study Population

A total of nine DM1 patients (6 females and 3 males) retrieved from our database were analyzed and matched for sex and age with nine controls. The median age at onset of DM1 in our patients was 20 (IQR 18–35) years, and the median age at the day of the examination was 36 (IQR 29–44) years. As required by the inclusion criteria, all of them are affect by DM1 and the length of the CTG repeat mutation ranged from 300 to 1,000 base pair.

The median age of our controls at the day of the examination was also 36 (IQR 29–44) years.

Raw data and distribution characteristics of our nine DM1 patients are reported in Table 1. Raw data of our controls are shown in Table 2.

**Table 1:** Raw clinical, genetic, and imaging data of our study population.

	**Anamnestic data**		**Genetic test**	**Neuromuscular assessment**			**Cardiological assessment**					**CMR assessment**			
	**Age at onset (y, range)**	**Age at CMR (y, range)**	**Length (bp)**	**MRC**	**MIRS^†^**	**Myotonia**	**Heart rate (bpm)**	**PR (ms)**	**QRS (ms)**	**QTc (ms)**	**EF (%)**	**GCS (%)**	**SCS (%)**	**GECV (%)**	**SECV (%)**
Patient 1	35–39	50–54	460	103	4	Absent	71	160	108^*^	450^*^	68	−16. 5	−24.0	43.2	39.2
Patient 2	25–29	25–29	630	130	5	Absent	73	140	102^*^	415	64	−16. 7	−15.6	38.4	37.6
Patient 3	15–19	20–24	300	130	2	Absent	70	233^*^	102^*^	439	65	−21. 7	−24.2	27.2	26.9
Patient 4	35–39	40–44	1,000	116	4	Absent	73	142	82	470^*^	67	−21. 3	−24.4	29.3	30.1
Patient 5	35–39	50–54	320	112	4	Absent	69	225^*^	138^*^	447^*^	52	−14. 0	−17.9	36.8	39.0
Patient 6	20–24	35–39	350	128	2	Absent	65	186	110^*^	445^*^	70	−20. 1	−22.5	27.5	29.0
Patient 7	10–14	30–34	680	112	3	Absent^∧^	69	194	105^*^	417	56	−20. 5	−20.3	31.1	30.5
Patient 8	10–14	25–29	680	130	2	Absent^∧^	71	174	112^*^	410	64	−19. 1	−18.7	32.6	34.1
Patient 9	15–19	35–39	490	126	2	Absent^∧^	73	144	84	402	56	−13. 9	−14.7	32.3	31.9
Median	20	36	490	126	3	–	71	174	105	439	64	−19.1	−20.3	32.3	31.9
IQR	18–35	29–44	350–680	112–130	2–4	–	69–73	144–194	102–110	415–447	56–67	−20.5 to −16.5	−24.1 to −17.9	29.3–36.8	30.1–37.6
Min-max	14–39	24–53	300–1,000	103–130	2–5	–	65–73	140–233	82–138	402–470	52–70	−21.7 to −13.9	−24.4 to −14.7	27.2–43.2	26.9–39.2

*CMR, cardiac magnetic resonance; MRC, medical research council scale; MIRS, muscular impairment rating scale; PR interval normal value <200 ms; QRS duration normal value <100 ms; QTc normal values ≤ 430 ms in female or ≤ 450 ms in male; EF left ventricular ejection fraction normal value >50%; GCS, global circumferential strain; SCS, septal circumferential strain; GECV, global extracellular volume; SECV, septal extracellular volume*.

* *values out of the normal range*.

∧ *patients on mexiletine*.

† *indicates that the three patients in our sample who had no symptomps of myotonia but were also under mexiletine (anti-dystonic drug)*.

**Table 2:** Raw imaging data of our control group.

	**Age at CMR (y, range)**	**CMR assessment**	
		**GCS (%)**	**SCS (%)**
Patient 1	50–54	−17. 7	−27. 6
Patient 2	25–29	−21. 7	−24. 2
Patient 3	20–24	−23. 4	−27. 0
Patient 4	40–44	−21. 3	−16. 9
Patient 5	50–54	−22. 5	−22. 6
Patient 6	35–39	−22. 7	−28. 3
Patient 7	30–34	−19. 3	−18. 8
Patient 8	25–29	−21. 3	−18. 3
Patient 9	35–39	−23. 9	−26. 7
Median	36	−21.7	−24.2
IQR	29–44	−22.7 to −21.3	−27.1 to −18.8
Min-max	24–53	−23.9 to −17.7	−28.3 to −16.9

*CMR, cardiac magnetic resonance*.

### Neuromuscular Assessment

The median value of MRC scale in DM1 patients was 126 (IQR 112–130) points with a distribution that ranged from 103 to 130 points. A negative correlation between MRC scale and the age at the examination was found (ρ = −0.809, *p* = 0.008).

Concerning the MIRS scale, the median score was 3 (IQR 2–4) points with a distribution which ranged from 2 to 5 points. In this case, a positive correlation was found between MIRS scale and the age of onset of DM1 in our patients (ρ = 0.687, *p* = 0.041).

In all DM1 patients clinical myotonia was absent at the day of the examination.

Moreover, no correlations were found between the genetic test and all the others parameters collected.

### Cardiological Assessment

The median heart rate was 71 (IQR 69–73) bpm with a distribution which ranged from 65 to 73 bpm. This value showed a very strong, negative correlation (ρ = −0.821, *p* = 0.007) with PR (median 174, IQR 144–194; range 140–233) ms and (ρ = −0.687, *p* = 0.041) with QRSD (median 105, IQR 102–110; range 82–138) ms.

The median value of QTc was 439 ms (IQR 415–447; range 402–470) ms, and positive correlations were found with the age of onset (ρ = 0.785, *p* = 0.012) and the age at the examination (ρ = 0.667, *p* = 0.05).

### Imaging Assessment

Patients' global CS (median −19.1%, IQR −20.5%, −16.5%; range −21.7 to −13.9%) was lower (*p* = 0.011) than that in controls (median−21.7%, IQR −22.7%, −21.3%; range −23.9 to −17.7%).

Patients' septal CS (median −20.3%, IQR −24.0%, −17.9%; range −24.4 to −14.7%) was not significantly different (*p* = 0.113) that in controls (median −24.2%, IQR −27.1%, −18.8%; range −23.9 to −17.7%). Septal CS negatively correlated with QTc (ρ = −0.733, *p* = 0.025).

Concerning ECV, global ECV was 32.3% (IQR 29.3, 36.8%) in DM1 patients with values that ranged from 27.2 to 43.2%; while septal ECV was 31.9% (IQR 30.1, 37.6%) with values which ranged from 26.9 to 39.2%. Moreover, the global ECV obtained in our DM patients was significantly (*p* = 0.008) higher than that (25.6%, IQR 19.9–31.9%) reported in literature (28) for healthy subjects.

**Figure 1:**
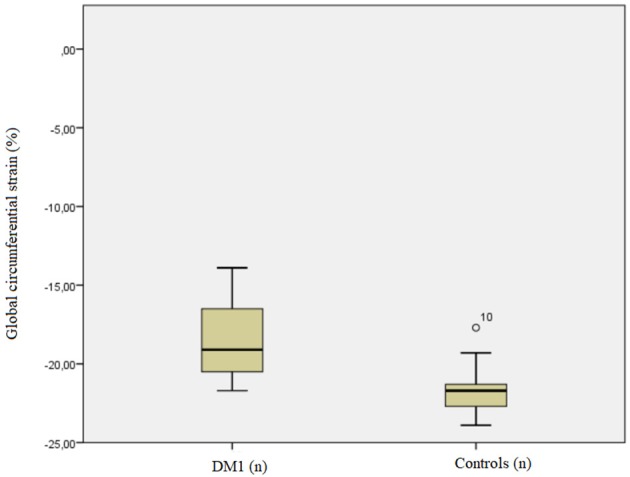
Boxplot showing the comparison of circumferential strain (CS) values in healthy controls vs. myotonic dystrophy (DM) patients.

**Figure 2:**
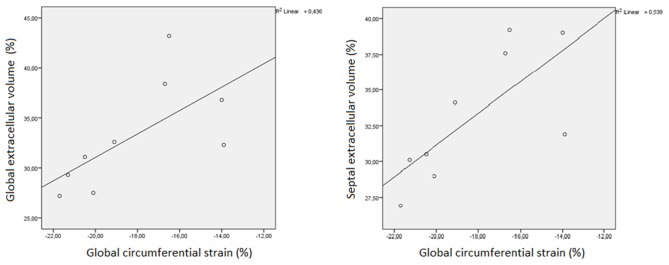
Linear regression plot representing the correlation between both global (GECV) and septal extracellular volume (SECV) and global circumferential strain (GCS) in myotonic dystrophy patients.

Global CS showed a strong, positive correlation with septal CS (ρ = 0.767, *p* = 0.016) and with both global (ρ = 0.733 *p* = 0.025) and septal ECV (ρ = 0.767, *p* = 0.016). A very strong positive correlation was observed between global ECV and septal ECV (ρ = 0.983, *p* < 0.001). Further details are shown in Figures 1, 2.

Albeit not statistically significant, a positive correlation was found between global ECV and the MIRS score (ρ = 0.621, *p* = 0.74).

## Discussion

The aim of our study was to evaluate ECV and strain on CMR in myotonic DM1 patient and appraise whether they could be viable imaging-biomarkers of subclinical cardiac pathology. Moreover, the secondary aim was to evaluate if there were some associations between imaging parameters and data stemming from clinical assessment and laboratory testing.

Thus, while at least some of the results (e.g., correlation between muscle impairment and the age of onset or between the ECG values) were expected or lacking clinical relevance, there are instead some main CMR findings that we believe might be useful to evaluate the subclinical condition of DM1 patients which we will discuss below.

Our global CS values were significantly lower in patients than in controls (*p* = 0.011), suggesting that contractility seems to be impaired in patients even in the absence of clinical dysfunction. The lack of a significant difference for septal CS between patients and controls may be due to the small population involved in the study. Indeed, segmental CS is more subject to error than global CS, since it depends both on contouring and reference points identification.

We compared ECV of our patients with that of healthy subjects reported in the meta-analysis published by Sardanelli et al. (28). The authors obtained a pooled ECV of 25.6% (IQR 19.9%−31.9%), resulting significantly lower (*p* = 0.008) compared to our ECV value of 32.3% (IQR 29.3, 36.8%). Therefore, we hypothesize that there may be a certain degree of subclinical diffuse fibrosis in the left ventricle of DM1 patients. A similar finding was reported by Schmacht et al. (29) on a population of myotonic dystrophy type 2 patients. The authors showed an increased ECV in positive LGE patients, confirming focal fibrosis, but also an increased ECV at the adjacent medial inferolateral segment without focal fibrosis detected by LGE. These results may indicate the occurrence of diffuse progressive fibrosis in these genetic conditions.

The positive correlation between both global and septal ECV and global CS may signify that contractile impairment in DM1 patients is due to fibrosis. The persistence of a significant correlation only at the septum may suggest that this phenomenon is more represented at septal level than in other areas. This agrees with previous pathological studies conducted on DM1 patients, which found the septum to be more prone to fibrosis than other left ventricular zones (9). Furthermore, a study by Garcia et al. (15) states that impaired strain obtained by echocardiography may be a predictor of adverse events in DM1 patients. We may thus hypothesize the same to be true for CMR-derived strain, since a strong correlation between CMR and echocardiographic strain values has already been proven (30).

Our study has some limitations, the most important being its small sample size and its retrospective design, which draw the need for prospective studies on bigger samples. However, we have to consider the prevalence of DM1 in the general population that makes, also in a reference center as our institution, DM1 patients difficult to find. Moreover, the fact that control subjects were lacking sequences for T1 mapping did not allow the calculation of ECV in this sample, thus leading to the need to rely on values obtained from external studies. Nevertheless, the normality values for ECV were assessed according to recommendations from the Society for Cardiovascular Magnetic Resonance (27). This may indeed present as a source of bias, even though ECV values, differently from T1 mapping results, have proven robust. Further prospective studies dedicated to the confirmation and in-depth analysis of the findings from this work should indeed address this limitation. Another further limitation is represented by the fact that this study only included the assessment of circumferential strain and did not appraise longitudinal or radial strain. Longitudinal strain was not calculated since not all patients had all the necessary acquisitions for its estimation, namely long-axis, 2-, 3-, and 4-chamber cine sequences. Radial strain was not included in the study because it has shown subpar reproducibility in previous analyses (31). Conversely, circumferential strain only requires short-axis cine sequences which were available in all patients and has shown satisfactory reproducibility in previous studies. Additionally, reproducibility of ECV was not appraised in this study due to the small study sample. Nevertheless, previous studies have observed an excellent inter-reader and inter-system reproducibility of ECV measurements (32).

We tested our sample ECV (median age 36 years old) with the one reported in the meta-analysis by Sardanelli et al. (28) which comprised subjects whose age ranged from 15 to 68. Given the slight but positive correlation between ECV and age, the point estimate provided by Sardanelli et al. (28) might overestimate the one for an ideal sample of 36 years old healthy subjects. Although this is a limitation to our study, we used this conservative approach since lower ECV values for controls would result into stronger statistical significance when tested against our patients ECV.

Our preliminary results suggest that in DM1 patients without apparent cardiac disease, the increase in ECV and decrease in CS may be taken into consideration as a trigger for follow-up and implementation of preventive measures. Our results are also consistent with those reported by Luetkens et al. (33) and Chmielewski et al. (34) who investigated the role of CMR in DM1. The first found lower myocardial strain and higher ECV in 13 DM1 patients compared to controls. The second reported similar results and found LGE presence to be independently associated with the occurrence of arrhythmic episodes. Therefore, we believe physicians dealing with DM1 patients may take into consideration CMR as an early screening tool to identify initial cardiac involvement in this condition.

## Data Availability Statement

The raw data supporting the conclusions of this article will be made available by the authors, without undue reservation, to any qualified researcher.

## Ethics Statement

The studies involving human participants were reviewed and approved by Comitato etico Ospedale San Raffaele, Milano, Italy. Written informed consent for participation was not required for this study in accordance with the national legislation and the institutional requirements.

## Author Contributions

All authors listed have made a substantial, direct and intellectual contribution to the work, and approved it for publication.

### Conflict of Interest

KC was employed by company Siemens Medical Solutions USA Inc. The remaining authors declare that the research was conducted in the absence of any commercial or financial relationships that could be construed as a potential conflict of interest.

## Abbreviations

DM1: myotonic dystrophy type 1
CMR: cardiac magnetic resonance
LGE: late gadolinium enhancement
ECV: extracellular volume
cTnT: cardiac Troponin T
MRC: medical research council
MIRS: muscular impairment rating scale
CS: cardiac strain.
